# Long-Term Clinical Outcomes of Radical Prostatectomy Versus Image-Guided and Intensity-Modulated Radiation Therapy for Prostate Cancer: A Retrospective and Comparative Study

**DOI:** 10.1155/aiu/6412793

**Published:** 2025-03-21

**Authors:** Tomoyuki Shimabukuro, Tanaka Hidekazu, Tanabe Masahiro, Takanori Tokunaga, Kosuke Shimizu, Nakanori Fujii, Keita Kobayashi, Hiroshi Hirata, Koji Shiraishi

**Affiliations:** ^1^Department of Urology, Graduate School of Medicine, Yamaguchi University, Ube, Yamaguchi, Japan; ^2^Department of Urology, Ube Central Hospital, Ube, Yamaguchi, Japan; ^3^Department of Radiation Oncology, Graduate School of Medicine, Yamaguchi University, Ube, Yamaguchi, Japan; ^4^Department of Radiology, Graduate School of Medicine, Yamaguchi University, Ube, Yamaguchi, Japan

**Keywords:** acute adverse events, IMRTG, long-term outcomes, prostate cancer, radical prostatectomy

## Abstract

**Background and Objective:** The optimal definitive treatment for localized prostate cancer (PCa)—radical prostatectomy (RP) or intensity-modulated radiation therapy with image guidance (IMRTG) remains controversial. This study compares the long-term clinical outcomes of RP and IMRTG in patients with PCa.

**Methods:** We retrospectively analyzed 884 consecutive PCa patients over 25 years. Among them, 610 (69%) underwent RP, while 274 (31%) received IMRTG starting in 2011. The primary objective was to comprehensively assess both treatment modalities.

**Results:** The median age was 68 years in the RP cohort and 73 years in the IMRTG cohort. The median operation time for RP was 4.11 h, with nerve-sparing procedures performed in 45% of cases. Median blood loss was 310 mL, the urinary incontinence rate was 13%, and the median hospital stay was 14 days. In the RP cohort, 46 complications (28%) occurred, including a Grade 4 rectal injury and a Grade 4 wound insufficiency. In the IMRTG cohort, over 80% of patients experienced radiation-induced urological complications, with 11% reporting Grade 2 adverse effects. During a median follow-up of 98 months, there were 79 all-cause deaths and 7 PCa-specific deaths. The 15-year overall survival (OS) rates were 80.9% for RP and 58.3% for IMRTG; however, this difference was not significant in the multivariate analysis, likely due to a higher proportion of high-risk disease in the IMRTG cohort. Approximately 45% of all deaths were attributed to malignant tumors.

**Conclusions:** This long-term retrospective study provides valuable insights into the comparative effects of RP and IMRTG on OS in PCa patients. Both treatments are associated with distinct adverse events, complications, and impacts on urinary continence and sexual function, highlighting the importance of individualized clinical decision-making.

## 1. Introduction

Definitive treatments for clinically localized prostate cancer (PCa) traditionally included radical prostatectomy (RP) and radiation therapy (RT) [1–3]. However, the absence of randomized controlled trials directly comparing their efficacy poses a challenge for clinicians and patients in making informed treatment decisions. Additionally, the impact of acute adverse events, surgical complications, and their effects on urinary continence, sexual function, and long-term oncological outcomes remains inadequately understood. Current clinical guidelines endorse both treatment options, further complicating the selection of the optimal approach.

Given this uncertainty, a thorough evaluation of treatment outcomes is crucial. Analyzing our institutional experience with RP and RT may yield valuable insights into their respective clinical efficacies, associated adverse events, and alternative strategies for PCa management. This study aims to comprehensively assess the effectiveness of RP and RT within our patient cohort while closely examining treatment-related adverse events. Furthermore, we investigate their potential impact on key oncological endpoints, including long-term overall survival (OS) and PCa-specific survival (PCSS). The primary objective of this study is to provide a rigorous comparison of both surgical and radiological treatments, offering insights to guide clinical decision-making. Based on 25 years of experience, this study represents the longest comparative analysis of oncological outcomes between these two treatment modalities and aims to contribute meaningful evidence to daily clinical practice.

## 2. Patients and Methods

### 2.1. Study Patients

Between January 2000 and December 2019, we identified 1278 patients with pathologically diagnosed PCa at Yamaguchi University Hospital and Ube Central Hospital. We evaluated a consecutive series of 610 patients who underwent RP (RP cohort) and 274 patients treated with intensity-modulated RT with image guidance (IMRTG cohort), with follow-up data collected until September 2024. Among the RP patients, 267 (43.8%) underwent open retropubic RP (ORRP), while 343 (56.2%) underwent robot-assisted RP (RARP). The primary aim of this study is to provide a comprehensive assessment of both surgical and radiological treatments.

### 2.2. Eligibility Criteria

Patients eligible for RP met the following criteria:

• Age ≤ 75 years with a life expectancy of at least 10 years
• Performance status of 1 or lower
• Initial PSA level < 20 ng/mL
• Clinical stage T2 or lower (N0M0)

Patients with uncontrolled glaucoma, severe heart disease, intracranial lesions, or obstructive pulmonary disease were excluded. For low-to intermediate-risk cases, MRI findings were considered, and nerve-sparing techniques were actively utilized. In high-risk cases, an extended RP, including extended lymph node dissection (covering internal and external iliac, obturator, and common iliac lymph nodes), were performed.

### 2.3. Marker Implantation and IMRTG

Radio-opaque fiducial gold markers (three markers per patient—two at the base and one at the apex of the prostate) were inserted using an introducer, guided by X-ray fluoroscopy and transrectal ultrasonography [4]. Following marker placement, standard IMRTG plus androgen deprivation therapy (ADT) was administered, delivering a prostate boost radiation dose of 74–78 Gy. ADT duration was determined based on the D'Amico risk classification: no ADT for low-risk patients, 6 months of ADT for intermediate-risk patients, and 24 months of ADR for high-risk patients. A 5-mm planning target volume (PTV) margin, including the rectal direction, was used for all IMRTG cases, enabled by the placement of gold markers. On-board imaging (OBI) was conducted daily, with cone-beam computed tomography (CBCT) performed daily for the first five treatments and at least once per week thereafter.

### 2.4. Assessment of Symptoms, Functions, and Adverse Events

Patients were assessed at baseline and again at discharge or 1 month postoperatively. Assessment tools included the Expanded PCa Index Composite (EPIC) [5], the International Prostate Symptom Score (IPSS), and the Short Form-8 Health Survey (SF-8). Collected data included operative time, nerve-sparing status, estimated blood loss, intraoperative and postoperative adverse events, urinary incontinence rates, and sexual function outcomes. Perioperative adverse events were classified using the Common Terminology Criteria for Adverse Events (CTCAE v5.0-JCOG). Sexual function was assessed using item 23 of the EPIC questionnaire and/or directly interviewed.

### 2.5. Diagnostic and Staging Procedures

All diagnostic and staging procedures adhered to the Clinical Practice Guideline for PCa issued by the Japanese Urological Association (JUA) [1]. Additionally, we followed the General Rules for Clinical and Pathological Studies on PCa, as outlined by the JUA, the Japanese Society of Pathology, and the Japan Radiological Society [6]. Postsurgical biochemical recurrence (BCR) was defined as a serum prostate-specific antigen (PSA) concentration ≥ 0.2 ng/mL, confirmed with a subsequent measurement [7]. Postradiotherapy BCR was defined as an increase of 2 ng/mL or more above the nadir serum PSA concentration following treatment, with or without hormonal therapy [8]. Medical events during follow-up were documented and evaluated at intervals of 1–12 months.

### 2.6. PSA Doubling Time (PSADT) Calculation

PSADT was calculated using the natural logarithm of 2 (0.693) divided by the slope of the relationship between the logarithm of PSA values and the timing of PSA measurements for each patient [9]. PSADT is considered reflective of tumor DT.

### 2.7. Pathological Diagnoses

All biopsy specimens were processed according to the General Rule for Clinical and Pathological Studies on PCa [6]. Prostates were serially sectioned to evaluate extraprostatic extension and resection margins. Various staining techniques were used, including anti-PSA antibody, anti-S100 antibody, anti-podoplanin antibody (D2-40), and Elastica van Gieson staining. Pathological diagnoses were conducted by board-certified pathologists following the General Rules guidelines.

### 2.8. Data Collection

Patients data were collected from written or electronic medical records, telephone interviews, radiology reports, and pathology reports. The collected information encompassed patient demographics (age, performance status, comorbidity), clinical stage, Gleason scores, and serum PSA concentration. Eligible patients had a minimum follow-up period of approximately 5 years, with data collection continuing until September 2024.

### 2.9. Study Approval

Approval for this study was obtained from the Institutional Review Board of the Yamaguchi University School of Medicine (approval number: H2020-229) and the Ube Central Hospital (approval number: 70160502).

### 2.10. Statistical Analysis

Statistical analyses were carried out using StatView version 4.5 (SAS, Cary, NC, USA). The association between both cohorts and clinicopathological factors was assessed using the Chi-squared test for categorical variables and the Mann–Whitney *U* test for continuous variables. OS and PCSS rates were estimated using the Kaplan–Meier method with log-rank comparisons. Univariable and multivariable analyses predicting OS or PCSS were conducted using the Cox proportional hazards model.

A significance level of less than 0.05 (*p* < 0.05) was considered statistically significant.

## 3. Results

### 3.1. Demographic and Clinical Characteristics

Table 1 summarizes the baseline characteristics of the enrolled patients. The median age (interquartile range) was 68 (64–71) years for the RP cohort and 73 (67–76) years for the IMRTG cohort. Patients in the IMRTG cohort were older and had higher T stages, a greater proportion of intermediate- and high-risk disease, and elevated baseline PSA levels compared to the RP cohort.

The median follow-up duration was 98 (73–144) months for the RP cohort and 97.5 (84–118) months for the IMRTG cohort. BCR occurred in 25% of RP patients (median time to BCR: 27 months) and in 4% of IMRTG patients (median time to BCR: 82 months). The median time to metastatic disease was 19 months for the RP cohort and 82 months for the IMRTG cohort.

During the follow-up period, there were 55 (9.0%) cases of all-cause mortality and 7 (1.2%) prostate cancer-specific deaths in the RP cohort, compared with 24 (8.8%) all-cause deaths in the IMRTG cohort.

### 3.2. Perioperative Outcomes

Table 2 outlines the perioperative outcomes of the RP cohort. The median operation time was 4.11 h, with nerve sparing procedures performed in 45% of patients. The median blood loss was 310 mL, the urinary incontinence rate was 13.0%, and the median hospital stay was 14 days.

### 3.3. Acute Phase Complications

Table 3 summarizes the acute phase complications in the RP cohort, categorized according to the CTCAE v5.0-JCOG. In the ORRP cohort, 12 complications were observed (12/36, 33.3%), while 34 complications occurred in the RARP cohort (34/129, 26.4%). The ORRP cohort included a Grade 4 rectal injury, while the RARP cohort experienced a Grade 4 wound insufficiency.  Table 4 summarizes the adverse events in the IMRTG cohort. Over 90% of patients experienced radiation-induced urological complications, with 11% reporting Grade 2 adverse effects. Radiation-induced proctitis affected approximately 20% of patients, while the incidence of radiation-induced cystitis was relatively low at 3.6%. The incidence of ≥ Grade 2 acute phase complications was 20% in the RP cohort and 14.6% in the IMRTG cohort.

### 3.4. Sexual Function

**Figure 1:**
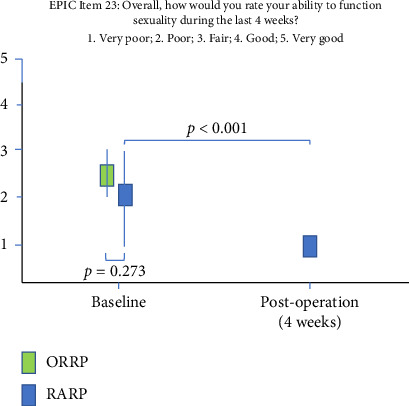
Baseline and postoperative overall sexual abilities. Key: ORRP, open radical retropubic prostatectomy; RARP, robot-assisted radical prostatectomy; EPIC, expanded prostate cancer index composite.

Figure 1 illustrates a significant decline in sexual function from baseline to 4 weeks postoperatively in the RARP group (*p* < 0.001).

### 3.5. OS

**Figure 2:**
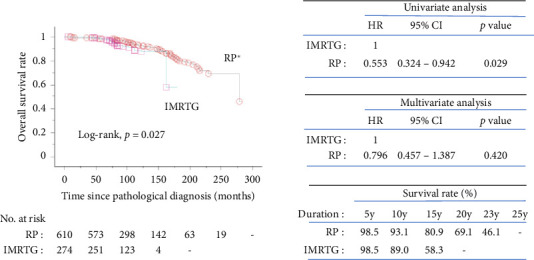
Kaplan–Meier overall survival rate plots of radical prostatectomy patients versus intensity-modulated radiation therapy with image guidance patients. Key: RP, radical prostatectomy; IMRTG, intensity-modulated radiation therapy with image guidance; HR, hazard ratio; 95% CI, 95% confidence interval; y, years. ⁣^∗^median survival months of RP = 277 months.

Figure 2 depicts Kaplan–Meier survival curves for OS. Cox regression analysis revealed a significant difference in OS between the RP and IMRTG cohorts in the univariate analysis, with the RP cohort demonstrating a substantially lower hazard ratio. However, this difference was not observed in the multivariate analysis, likely due to the IMRTG cohort having a higher proportion of high-risk disease at baseline.

The 10-year OS rates were 93.1% for the RP cohort and 89.0% for the IMRTG cohort, while the 15-year OS rates were 80.9% and 58.3%, respectively.

### 3.6. PCSS

**Figure 3:**
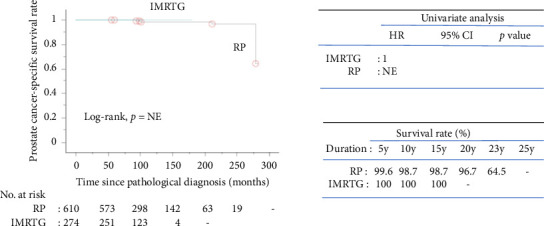
Kaplan–Meier prostate cancer-specific survival rate plots of radical prostatectomy patients versus intensity-modulated radiation therapy with image guidance patients. Key: RP, radical prostatectomy; IMRTG, intensity-modulated radiation therapy with image guidance; HR, hazard ratio; 95% CI, 95% confidence interval; NE, not estimable; y, years.

Figure 3 illustrates the Kaplan–Meier curves for PCSS. No mortality was observed in the IMRTG cohort, rendering the data nonestimable. The 10-year PCSS rates were 98.7% for the RP cohort and 100% for the IMRTG cohort, with identical rates observed at 15 years.

### 3.7. Causes of Death

Table 5 lists the causes of death in both cohorts. Approximately 45% of all deaths were attributed to malignant tumors, followed by heart diseases (19%) and lung diseases (15%).

## 4. Discussion

Recent longitudinal studies have shed light on the extended clinical outcomes of various treatment modalities for localized PCa [10]. Notably, the ProtecT trial utilized radiotherapy combined with neoadjuvant ADT for 3–6 months, employing three-dimensional conformal RT (3D-CRT) at 74 Gy in 37 fractions. In contrast, our study employed IMRTG at 78 Gy in 39 fractions, accompanied by ADT. Several treatment planning studies have consistently demonstrated the superiority of IMRT over 3D-CRT in minimizing radiation exposure to surrounding organs [11–13]. Furthermore, the integration of image-guided RT (IGRT) in our study enhanced treatment precision [4].

This study aimed to compare IMRTG and RP in patients with clinically localized or locally advanced PCa, focusing on clinical characteristics, acute adverse events, complications, urinary continence, sexual function, and long-term oncologic outcomes. Initially, the study included patients treated with 3D-CRT, brachytherapy, and heavy-ion therapy. However, to ensure a robust comparison of IMRTG and RP, we ultimately narrowed the cohort to 274 patients receiving IMRTG due to the limited number of patients in the non-IMRT treatment groups.

BCR was observed in 25% of RP patients (median time: 27 months) and 4% of IMRTG patients (median time: 82 months) (Table 1). Salvage treatments for biochemically recurrent PCa (BRPC) after RP included observation, external beam RT plus ADT, or ADT alone, depending on risk factors such as pathological Gleason score ≥ 8, PSADT at BCR ≤ 9 months, and time to BCR ≤ 12 months). In contrast, salvage treatments for BRPC after IMRTG included observation, ADT alone, or ADT combined with an androgen receptor signaling inhibitor. Distant metastases occurred in 1.8% (11/610) of RP patients (median time: 19 months) and 1.5% (4/274) of IMRTG patients (median time: 82 months) (Table 1). The management of metastatic disease followed Japanese clinical practice guidelines for metastatic castration-sensitive or castration-resistant PCa [1].

Table 2 summarizes the perioperative outcomes of the RP cohort. The median operation time was 4.11 h, and nerve sparing techniques employed in 45% of cases. Median blood loss was 310 mL, and urinary incontinence occurred in 13.0% of patients, with a median hospital stay of 14 days. In comparison, Yaxley et al. reported mean surgery times of 3.9 h for ORRP and 3.4 h for RARP, with average blood loss of 1338 mL for ORRP and 444 mL for RARP. The reported median hospital stays were 3.27 days for ORRP and 1.55 days for RARP [14]. In our study, estimated blood loss in the ORRP cohort was approximately three times higher than in the RARP cohort (*p* < 0.001), and the median hospital stay was significantly longer (*p* < 0.001). These findings are consistent with previous studies, except for hospital stay duration, which may vary due to differences in healthcare systems and insurance policies [15, 16].

Acute phase complications (Table 3) were reported in 33.3% (12/36) of ORRP patients and 26.4% (34/129) of RARP patients, including two Grade 4 adverse events. Novara et al. conducted a systematic review and reported a mean complication rate of 9%, with significantly lower blood loss and transfusion rates in RARP, although other complications were comparable between ORRP and RARP [15].

A prospective, controlled, nonrandomized trial comparing ORRP and RARP found that RARP modestly improved erectile function at 12 months postsurgery [17]. In our study, early sexual function (EPIC item 23) declined significantly 4 weeks postoperatively in the RARP cohort (*p* < 0.001) (Figure 1). However, due to the lack of postsurgical sexual function data for the ORRP cohort, a direct comparison was not feasible. Erectile function can improve for up to 3 years postsurgery [18], and we continue to assess this aspect. Notably, nerve-sparing technique were employed in 55.0% of RARP cases versus only 8.3% of ORRP cases. This reflects the robotic-assisted approach's enhanced visualization and precision, leading to better functional outcomes in urinary continence and erectile function, particularly in low-and intermediate-risk patients [19].

Table 4 summarizes radiation-induced complications in the IMRTG cohort. Over 80% of patients experienced radiation-induced urological complications, with 11% reporting Grade 2 adverse effects. Radiation-induced proctitis affected approximately 20% of patients, while the incidence of radiation-induced cystitis was relatively low at 3.6%. Our proctitis incidence was higher than in previous reports, likely due to stricter diagnostic criteria. Zelefsky et al. reported acute gastrointestinal (GI) toxicity (≥ Grade 2) at 3% and genitourinary (GU) toxicity at 37%, noting that acute toxicities often predict late toxicities [20]. A review by David et al. found 60-month cumulative incidences of GU toxicity (≥ Grade 2) at 33%, hematuria at 5%, urinary incontinence at 12%, and urinary retention at 24% [21]. Overall, the incidence of ≥ Grade 2 acute phase complications was 20% in the RP cohort and 15% in the IMRTG cohort, showing comparable outcomes between the two treatments.

The overall mortality rate was 8.9%, and the PCa-specific mortality rate was 0.8% over a median follow-up of 98 months. The ProtecT trial reported 15-year all-cause mortality at 21.7% and PCa-specific mortality at 2.7% [10]. Kaplan–Meier survival analysis (Figure 2) showed significant differences in OS between the RP and IMRTG cohorts in univariate analysis, with RP showing a lower hazard ratio. However, this significance diminished in multivariate analysis, likely due to higher-risk disease in the IMRTG cohort. A systematic review comparing radical surgery and radiotherapy in localized PCa consistently reported higher mortality rates with radiotherapy [22]. Our OS rates for radiotherapy and surgery were 89.0% and 93.1% at 10 years, and 58.3% and 80.9% at 15 years, respectively. Kaplan–Meier analysis for PCSS (Figure 3) showed 100% survival in the IMRTG cohort, rendering the data nonestimable. Our PCSS rates for radiotherapy and surgery were both 100% and 98.7% at 10 and 15 years, respectively, comparable to the ProtecT trial's PCSS rates of 97.1% and 97.8% for radiotherapy and surgery, respectively [10].

Table 5 outlines the causes of death in both cohorts. Approximately 45% of all deaths were attributed to malignant tumors, followed by heart and lung diseases, reflecting the aging nature of the patient population.

Several limitations must be acknowledged. First, this is a retrospective observational study with a moderate sample size. Second, outcomes may have been influenced by the involvement of multiple physicians and diverse treatment approaches. Third, imbalance in treatment group size, as RP was prioritized before IMRTG became widely available. Forth, the limited number of PCa-specific mortality events restricts comprehensive evaluation of PCSS. Lastly, the study does not specifically address long-term adverse effects, including radiation-induced urological or GI complications, urinary incontinence, and sexual dysfunction.

## 5. Conclusion

This long-term retrospective study provides comparative insights into RP and IMRTG outcomes in PCa patients. Both treatments resulted in adverse events, complications, and impacts on urinary continence and sexual function. Clinical decision-making should consider these factors, and continuous advancements in treatment techniques and post-treatment care are essential to improving patient outcomes.

## Figures and Tables

**Table 1:** Baseline demographic and clinical characteristics.

	All	RP	IMRTG	*p* value
	(*n* = 884)	(*n* = 610)	(*n* = 274)	
Age, y				< 0.001
< 65	217	183	34	
≥ 65	667	427	240	
Clinical T stage				< 0.001
≤ T2	793	574	219	
≥ T3	91	36	55	
Clinical N stage				0.246
0	873	603	270	
1	8	4	4	
x	3	3	0	
D'Amico risk group				< 0.001
Low-risk	119	91	28	
Intermediate-risk	369	279	90	
High-risk	395	239	156	
Baseline PSA status (ng/mL)				0.012
≤ 10.0	515	363	152	
10.1–20.0	245	175	70	
> 20.0	122	70	52	
PSA doubling time at BCR, month				0.014
≤ 9	64	62	2	
> 9	42	35	7	
Follow-up characteristics				
Median follow-up months	98.0	98.0	97.5	0.108
Median months to BCR	29.5	27.0	81.5	0.001
Median months to DM	54.0	19.0	82.0	0.129
Biochemical recurrence-no. (%)				
Present	163 (18.4)	152 (24.9)	11 (4.0)	< 0.001
Distant metastases-no. (%)				
Present	15 (1.7)	11 (1.8)	4 (1.5)	0.727
Mortality-no. (%)				
All-cause mortality	79 (8.9)	55 (9.0)	24 (8.8)	0.901
PCa-specific mortality	7 (0.8)	7 (1.2)	0	0.075

Abbreviations: BCR, biochemical recurrence; DM, distant metastases; IMRTG, intensity-modulated radiation therapy with image guidance; PCa, prostate cancer; PSA, prostate-specific antigen; RP, radical prostatectomy; y, years.

**Table 2:** Perioperative outcomes.

	All	ORRP	RARP	*p* value
	(*n* = 165)	(*n* = 36)	(*n* = 129)	
Operation time (hours)				0.067
Median	4.11	4.15	4.08	
IQR	3.39–5.01	3.48–5.13	3.35–5.01	
Nerve sparing technique				< 0.001
Yes	74	3	71	
No	91	33	58	
Bleeding volume (mL)				< 0.001
Median	310.0	680.0	217.5	
IQR	113.8–550.0	538.8–1326.3	101.0–410.0	
% of urinary incontinence				0.366
Median	13.0	17.1	13.0	
IQR	3.2–37.0	2.0–27.5	3.6–37.7	
Hospital treatment days				< 0.001
Median	14	18	14	
IQR	12–17	14–20	12–16	

Abbreviations: IQR, interquartile range; ORRP, open radical retropubic prostatectomy; RARP, robot-assisted radical prostatectomy.

**Table 3:** Acute phase complications of radical prostatectomy.

	ORRP (*n* = 36)					RARP (*n* = 129)				
	Grade 1	Grade 2	Grade 3	Grade 4	Grade 5	Grade 1	Grade 2	Grade 3	Grade 4	Grade 5
Wound insufficiency	1		2			5			1	
Rectal injury			2	1				2		
Urine leakage/urinoma			1			1	2	1		
Subcuraneous bleeding						1	1			
Muscle injury						1	1			
Hemorrhagic duodenal ulcer								2		
Epididymitis		2								
Fever			1				2			
Hypotention								1		
Difficult urination·Urinary retension		1					3			
Intervertebral disk herniation						1				
Post bleeding						1		3		
Paralytic ileus							1			
Acute sensorineural hearing loss							1			
Congestive lung								1		
Gout attack							1			
Headache						1				
Herpes zoster	1									

Abbreviations: ORRP, open radical retropubic prostatectomy; RARP, robot-assisted radical prostatectomy.

**Table 4:** Acute phase adverse events of IMRTG (*n* = 247).

Symptoms	Grade 1	Grade 2	Grade 3/4/5
Pollakisuria	137	15	
Urgency	3		
Dysuria	43	11	
Painful urination	19	1	
Painful bowel movement	16		
Radiation-induced proctitis	48	1	
Radiation-induced cystitis	9		
Radiation-induced dermatitis	3		
Radiation urethritis	1		
Complete urinary retention		1	
Anemia (upper intestine bleeding)		2	
Leukopenia		5	
Hangover	1		
Fatigue	11		
Hemorrhoid aggravation	4		
None	40		

Abbreviation: IMRTG, intensity-modulated radiation therapy with image guidance.

**Table 5:** Causes of deaths.

	RP		IMRTG (*n* = 24)
	ORRP (*n* = 47)	RARP (*n* = 8)	
Malignant tumors	23	3	9
Prostate cancer	7	0	0
Pancreatic cancer	3		1
Bladder cancer	3		
Gastric cancer	3	1	2
Liver cancer	2		2
Renal cell cancer			2
Esophageal cancer	1		
Lung cancer	1	1	1
Colon cancer	1		
Malignant mesothelioma		1	
Malignant lymphoma	1		1
Myelodysplastic syndrome	1		
Heart-related diseases	9	1	2
Heart failure	4		
Sudden death	2	1	2
Myocardial infarction	1		
Aortic stenosis	1		
Valve regurgitation	1		
Lung-related diseases	7		4
Aspiration pneumonia	4		1
Interstitial pneumonia	2		
Adult respiratory distress syndrome	1		3
Acute stroke	2		2
Progressive supranuclear palsy	1		
Neural disease	1		2
Renal failure		1	
Urinary tract infection	1		
Liver dysfunction	1		
Diabetes mellitus			1
Suicide		2	
Aging	1		2
Unknown	1	1	2

Abbreviations: IMRTG, intensity-modulated radiation therapy with image guidance; ORRP, open radical retropubic prostatectomy; RARP, robot-assisted radical prostatectomy; RP, radical prostatectomy.

## Data Availability

The data that support the findings of this study are not openly available due to reasons of sensitivity and are available from the corresponding author upon reasonable request.

## Nomenclature

BCR: Biochemical recurrence
EPIC: Expanded prostate cancer index composite
HR: Hazard ratio
IGRT: Image-guided radiation therapy
IMRT: Intensity-modulated radiation therapy
IMRTG: IMRT with image guidance
ORRP: Open retropubic radical prostatectomy
OS: Overall survival
PCa: Prostate cancer
PCSS: Prostate cancer-specific survival
PSA: Prostate-specific antigen
PSADT: PSA doubling time
RARP: Robot-assisted radical prostatectomy
RP: Radical prostatectomy
RT: Radiation therapy
